# Exploring the Early Time Behavior of the Excited States of an Archetype Thermally Activated Delayed Fluorescence Molecule

**DOI:** 10.1021/acs.jpclett.4c00030

**Published:** 2024-02-07

**Authors:** Larissa G. Franca, Andrew Danos, Rishabh Saxena, Suman Kuila, Kleitos Stavrou, Chunyong Li, Stefan Wedler, Anna Köhler, Andrew P. Monkman

**Affiliations:** † Department of Physics, Durham University, South Road, Durham DH13LE, United Kingdom; ‡ Soft Matter Optoelectronics and Bavarian Polymer Institute (BPS), University of Bayreuth, Universitätsstrasse 30, Bayreuth 95440, Germany

## Abstract

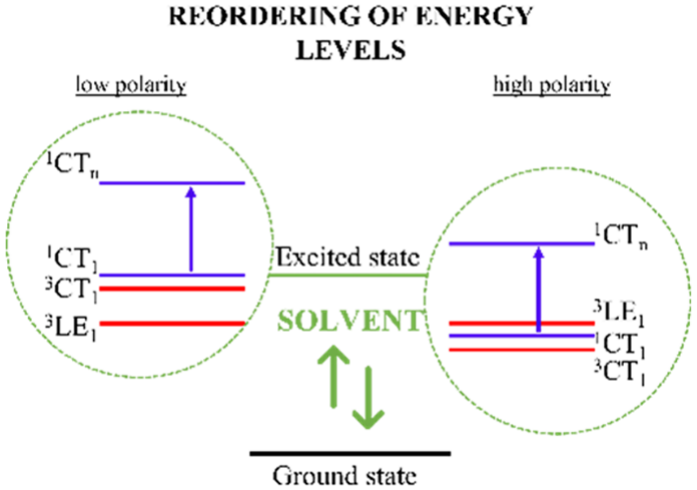

Optical pump–probe techniques allow for an in-depth study of dark excited states. Here, we utilize them to map and gain insights into the excited states involved in the thermally activated delayed fluorescence (TADF) mechanism of a benchmark TADF emitter **DMAC-TRZ**. The results identify different electronic excited states involved in the key TADF transitions and their nature by combining pump–probe and photoluminescence measurements. The photoinduced absorption signals are highly dependent on polarity, affecting the transition oscillator strength but not their relative energy positions. In methylcyclohexane, a strong and vibronically structured local triplet excited state absorption (^3^LE → ^3^LE_*n*_) is observed, which is quenched in higher polarity solvents as ^3^CT becomes the lowest triplet state. Furthermore, ultrafast transient absorption (fsTA) confirms the presence of two stable conformers of **DMAC-TRZ**: (1) quasi-axial (QA) interconverting within 20 ps into (2) quasi-equatorial (QE) in the excited state. Moreover, fsTA highlights how sensitive excited state couplings are to the environment and the molecular conformation.

The prospect for future energy-efficient and low-cost organic light-emitting diodes (OLEDs) has been greatly improved by the introduction of thermally activated delayed fluorescence (TADF) molecules.^1,2^ Due to their ability to convert dark triplet excitons into emissive singlets without using rare/expensive heavy metals, TADF molecules can reach up to 100% internal quantum efficiencies while avoiding many of the problems associated with phosphorescent OLEDs.^3^

For TADF materials, minimizing the energy gap between the singlet and triplet excited states (Δ*E*_ST_) is essential to facilitate triplet harvesting through thermally assisted reverse intersystem crossing (rISC).^4,5^ A typical design strategy for these molecules consists of an electron donor (D) and an acceptor (A) unit linked to each other.^6^ This molecular structure creates spatial separation between the highest occupied and the lowest unoccupied molecular orbitals (HOMOs and LUMOs, respectively), which leads to low electron correlation energy and the required small Δ*E*_ST_.^7,8^ Moreover, the interaction between the D and A units promotes the formation of excited states with charge transfer (CT) character. The presence of multiple states of different orbital character plays an important role in facilitating the spin-flip from the triplet to singlet excited states. As spin–orbit coupling (SOC) between ^1^CT and ^3^CT is formally forbidden, the rISC mechanism is mediated by an energetically close local triplet (^3^LE) state, where vibronic coupling with ^3^CT allows the upconversion to the ^1^CT states.^9^ Thus, the small energy gap between the ^3^LE and CT (both ^1^CT and ^3^CT) increases rISC rates and, consequently, enhances TADF efficiency.^10^

Due to the CT character of their excited states, the emissive properties of D–A TADF molecules are highly dependent on the environment.^11^ However, given the different sensitivity of CT and LE states to the specific environmental properties, e.g., polarity/polarizability, a change in the relative energy ordering of the TADF molecular excited states is expected in different environments.^12,13^ In this context, three different scenarios for energy ordering can be achieved:^9,14^ (i) the ^3^LE state is lower in energy than the CT states (both ^1^CT and ^3^CT); (ii) the three states involved (^3^LE, ^1^CT, and ^3^CT) are energetically aligned with each other; (iii) the ^3^LE state has higher energy than the CT states (both ^1^CT and ^3^CT). Thus, not only does Δ*E*_ST_ play an important role in the dynamic mechanisms of rISC but also the relative positions of the excited states.^15^

Photoluminescence emission and lifetimes are often used to study the excited state of TADF molecules.^16,17^ These methodologies rely heavily on the emissive excited states and do not easily give information about the dark excited states involved in the TADF process such as ^3^CT.^18,19^

Herein, we used photoinduced absorption techniques to map the excited states of **DMAC-TRZ** in different environments, unconstrained by the limitation of focusing only on emissive states. **DMAC-TRZ** was chosen as a TADF molecule benchmark, as considerable work has already been reported for this molecule.^12,20−24^ We identified both the transitions and the nature of the electronic excited states involved in rISC by combining quasi-CW and transient photoinduced absorption (both flash photolysis and ultrafast) with standard photoluminescence measurements. Moreover, we directly observed a reorganization of excited states with increasing solvent polarity, which leads to quenching of the T_1_→ T_*n*_ induced absorption when ^3^CT becomes the lowest triplet excited state. Because of its nature and significantly longer lifetimes, ^3^CT is more susceptible to quenching through nonradiative decays in line with the energy gap law. In ultrafast transient absorption, we also reveal evidence that rapid solvent reorganization strongly affects the oscillator strength of the singlet excited state transitions.

For reference, linear absorption and emission as a function of solvent are given in Figure 1, highlighting the CT characteristics of the molecule by increasing the solvent polarity. It is worth mentioning that, despite the well-structured vibronic peak in the emission spectrum of MCH, the excited states exhibit a mixture of ^1^LE/^1^CT character. This result has been explored in previous studies, where these findings were supported by detailed quantum mechanical calculations.^25−27^ To study the energy ordering of the electronic excited states in **DMAC-TRZ** (molecular structure shown in Figure S1), we first performed quasi-CW photoinduced absorption (PIA) measurements on concentrated (0.8 mM) solutions. All measurements were made at room temperature in an oxygen-free solution. Using PIA measurements, we can probe both emissive and nonemissive excited states, which broadens insight into the excited state landscape beyond the information obtained from emission spectroscopy alone. The apparatus is described in the SI, and it allows absorbance features of the excited states (including absorbance features of triplet excited states, photoinduced by the 375 nm pump beam) to be detected through changes in transmission of an overlapping probe beam. The phase of the lockin signal also allows us to broadly distinguish spectral features arising directly in phase with the pulsed pump beam as well as those occurring indirectly (out of phase).

**Figure 1:**
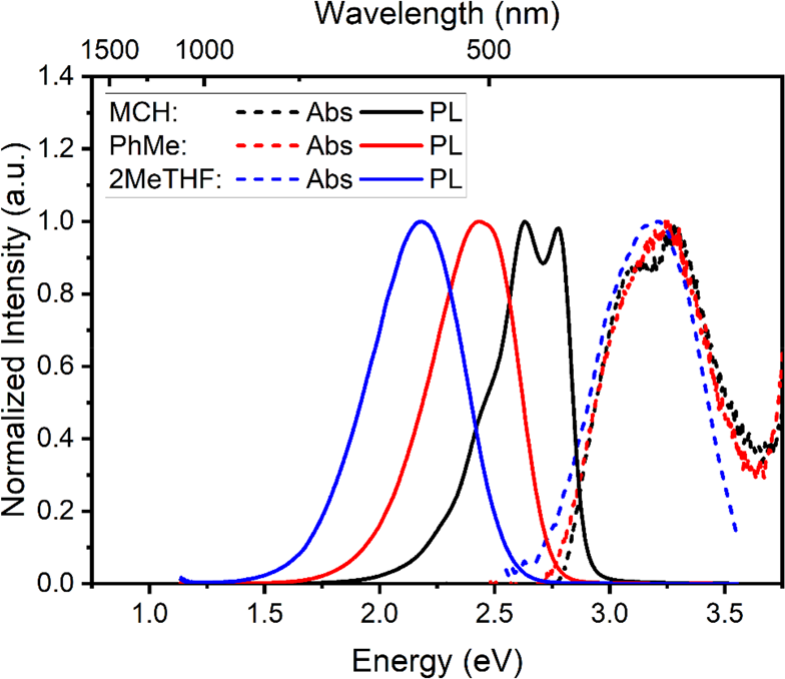
Normalized absorption (dashed lines, 20 μM) and emission spectra (solid lines, 0.8 mM) of **DMAC-TRZ** in methylcyclohexane (MCH), toluene (PhMe), and 2-methyltetrahydrofuran (2MeTHF). λ_exc_ = 365 nm.

Figure 2 shows the PIA spectra of **DMAC-TRZ** in three different solvents: methylcyclohexane (MCH), toluene (PhMe), and 2-methyltetrahydrofuran (2MeTHF) at 0.8 mM. At this relatively high concentration, necessary for achieving good PIA signal-to-noise ratio, we additionally performed time-resolved photoluminescence measurements. These gave similar spectra and lifetimes as in very low concentration solutions (Figure S2),^12^ indicating no significant dimer/excimer formation occurs at the higher concentrations (Figures S3 and S4). The PIA spectra of **DMAC-TRZ** in all solvents gave signals in phase (*X*) as well as out of phase signal (*Y*) with respect to the excitation pulse train (in this case, *f* = 173 Hz). The signals observed in phase arise from transitions of short lifetime (relative to 1/*f* of the excitation pulse train, i.e., around 6 ms), whereas the out of phase signals are from long lifetime transitions (usually related to triplet transitions). The PIA spectra for all solutions showed a positive Δ*T*/*T* feature in the *X* component at high energies (Figure 2), which is observed to spectrally overlap with the photoluminescence (PL) spectra in each solvent, as shown in Figure 1. This is ascribed to either the direct collection of a photoluminescence signal or stimulated emission (SE) of **DMAC-TRZ**, which follows the PL solvatochromism (as seen in Figure 1). At higher energy above the PL/SE, we observe a Δ*T*/*T* contribution from photobleaching of the ground state transition, Figure 2b. Both PL/SE and ground state bleaching appear weaker and less well-resolved in MCH. This could be attributed to the strong overlap of both bands and the fact that the emission in MCH is very close to our detection limit of the hardware, ∼400 nm, effectively masking the GSB signal in this case. Induced absorption (−Δ*T*/*T*) was also observed in both the *X* and *Y* components. In both MCH and PhMe, a broad negative-induced absorption in the X channel indicates an overlap of signals originating from various electronic transitions.

**Figure 2:**
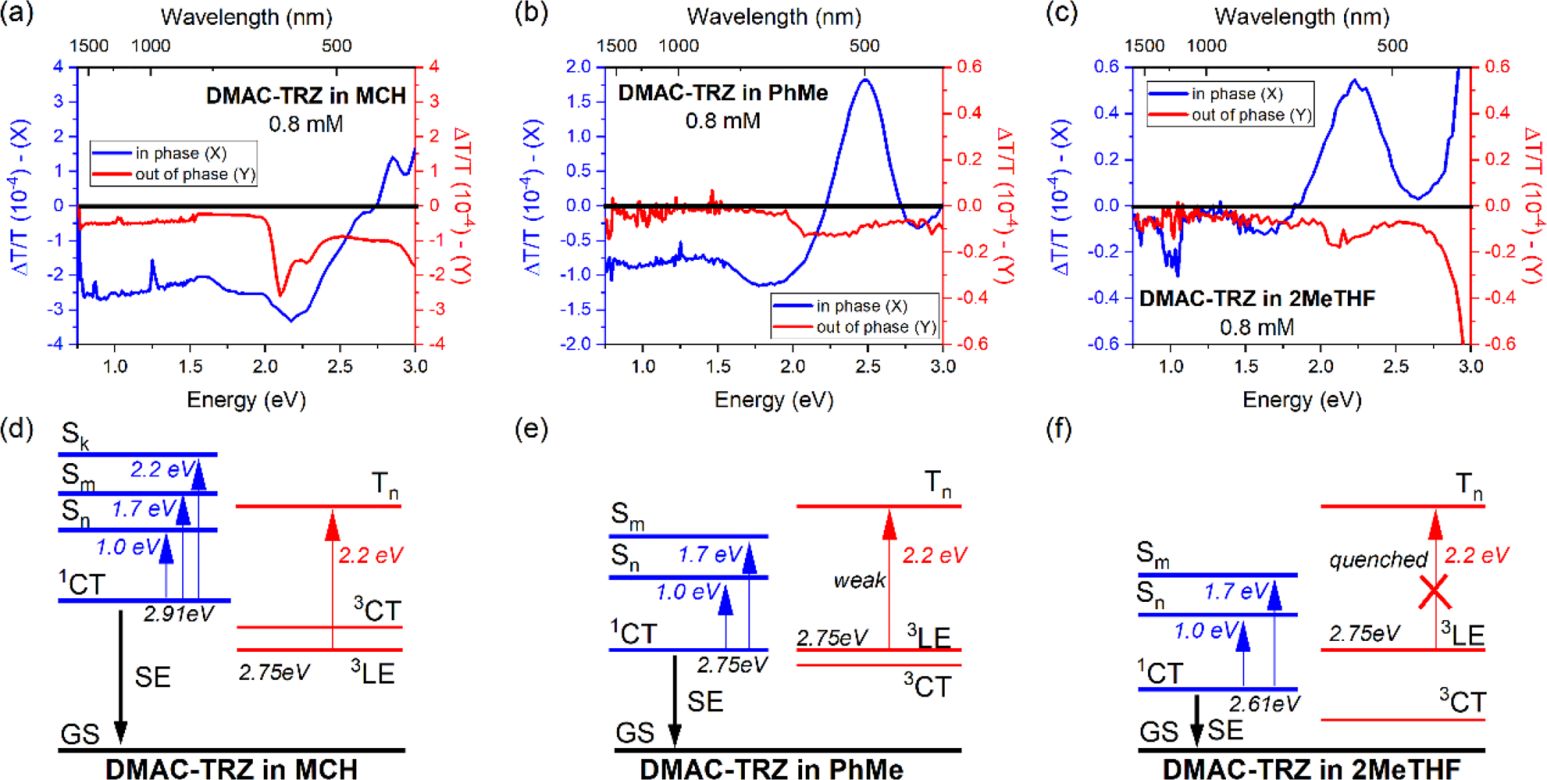
Top panels: Quasi-CW photoinduced absorption of **DMAC-TRZ** in (a) methylcyclohexane (MCH), (b) toluene (PhMe), and (c) 2-methyltetrahydrofuran (2MeTHF) solutions at 0.8 mM. λ_exc_ (pump) = 375 nm. Bottom panels: Proposed excited state energy diagram, representing the spectra obtained by the quasi-CW photoinduced absorption of **DMAC-TRZ** in different solvents.

The induced absorption in both *X* and *Y* channels becomes significantly weaker with increasing solvent polarity. Higher polarity solvents decrease the oscillator strength (*f*) of CT transitions, indicating that the PIA bands observed here in the *X* channel are most likely from the first excited CT states to upper high energy CT states (i.e., ^1^CT_1_ → ^1^CT_*n*_). Two induced bands (∼1.75 and ∼1.0 eV) have much lower intensity in higher polarity solvents (2MeTHF), but their band positions remain the same. As the transition energies are unaffected in all solvents, this confirms that these two transitions, (S_1_ → S_*n*_ and S_1_ → S_*m*_) are between states having the same character, i.e., LE to LE or CT to CT but not LE to CT or CT to LE (which would be affected differentially by solvent polarity, thus shifting the band position). In the *Y* channel, a well-defined structured induced absorption band around 2.2 eV is observed in MCH solution, indicating a state with strong local character and with long lifetime, a transition likely coming from the lowest triplet excited state of the molecule (T_1_ → T_*n*_). As recently demonstrated by us,^26^**DMAC-TRZ** in nonpolar solvent displays two distinct stable excited state conformers: a quasi-axial (QA) and a quasi-equatorial (QE) conformer (Figure S5). By exciting at 3.30 eV (375 nm, the pump wavelength), both the QA and QE can be independently populated. Also, Dexter energy transfer from the QE to the QA triplet excited state populates the lowest triplet excited state in MCH, as shown to be the QA triplet excited state in this scenario.^26^ Thus, we assign the strong vibronic transition observed in the Y channel (T_1_ → T_*n*_) to ^3^LE_1_ → ^3^LE_*n*_ of the QA conformer. This is in good agreement with our calculations, which demonstrates the triplet excited state of QA has a mixed LE/CT character with stronger LE component, while QE exhibits pure CT character.^26^

As solvent polarity increases, this structured long-lived signal is effectively quenched, e.g., in 2MeTHF, where we see a little excited state population in the *Y* channel (longer lifetimes). The higher polarity solvents not only destabilize the QA conformer^26^ but also significantly increase the CT character and relax its energy.

We then performed transient photoinduced absorption measurements (nsTA) on the same solutions to confirm the presence and help in the assignment of these transitions observed in the CW-PIA spectra (Figure 3). In this technique, the lockin detection of CW-PIA is replaced with a fast photodiode and oscilloscope in order to monitor the kinetics of a specific transition directly. Measurements were collected both with the white light probe beam off (pump only background, also corresponding to PL) and with both pump and probe beams overlapping (TA), allowing us to obtain both photoluminescence and the induced absorption lifetime, respectively. Decay traces obtained by probing different energies of MCH, PhMe, and 2MeTHF solutions are shown in Table 1, with the selected energies informed by the previous CW-PIA spectra. Note that, in some measurements, the oscilloscope vertical scale was deliberately chosen in a way that saturates the early signal (corresponding to prompt fluorescence (PF)), in order to achieve adequate resolution of the delayed signal.

**Figure 3:**
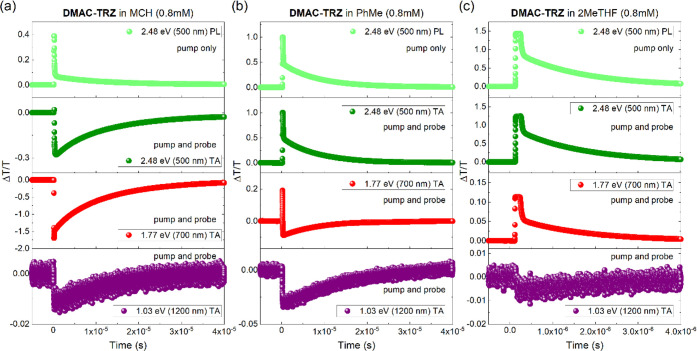
Transient photoinduced absorption of **DMAC-TRZ** in (a) MCH, (b) PhMe, and (c) 2MeTHF. The measurements were collected at 2.48 eV (500 nm), with the pump only (PL) and pump and probe (TA), at 1.77 eV (700 nm) with the pump and probe (TA), and at 1.03 eV (1200 nm) with the pump and probe (TA). λ_exc_ (pump) = 355 nm.

**Table 1:** Lifetimes Obtained from the Fitting of Transient Photoinduced Absorption of **DMAC-TRZ** in MCH, PhMe, and 2MeTHF Solutions at 0.8 mM (Figures S6–S11)^a^

**DMAC-TRZ**		MCH	PhMe	2MeTHF
2.48 eV/500 nm	PL	13.3 μs	8.6 μs	1.5 μs
	TA	11.6 μs (91.4%)/0.14 ms (8.6%)	8.2 μs	1.5 μs
2.06 eV/600 nm	PL		8.6 μs	1.6 μs
	TA	11.5 μs (92.2%)/0.16 ms (7.8%)	8.4 μs (93%)/0.12 ms (7%)	1.5 μs
1.77 eV/700 nm	PL			1.5 μs
	TA	13.2 μs	8.1 μs	1.5 μs
1.55 eV/800 nm	PL			1.5 μs
	TA			1.6 μs
1.03 eV/1200 nm	PL			
	TA	11.5 μs	9.1 μs	2.9 μs
Time Resolved Photoluminescence Decay				
^1^CT (eV)		2.91	2.75	2.61
Δ*E*_ST_ (eV)^b^		0.16	0	–0.14
PF		13.6 ns	21.4 ns	24.6 ns
DF		20.1 μs	8.4 μs	1.5 μs
*k*_F_ (s^–1^) × 10^7^		6.03	2.05	1.69
*k*_ISC_ (s^–1^) × 10^7^		1.33	2.63	2.37
*k*_rISC_ (s^–1^) × 10^4^		6.05	27.1	163

a The lifetime and the estimated rates were obtained from the kinetics of the photoluminescence decay (λ_exc_ = 355 nm) (Figure S4). Thus, note that the conventional sign and value of Δ*E*_ST_ has developed in this field such that negative values imply that the energy of the ^3^LE state is above the ^1^CT excited state and the ^3^CT is the lowest triplet excited state.

b Δ*E*_ST_ = ^1^CT – ^3^LE.

Lifetimes estimated from the fitting of nsTA measurements are in good agreement with the lifetimes estimated from the fitting of time-resolved photoluminescence decay (Figure S4). The broad negative band (−Δ*T*/*T*) observed in the *X* component of PIA (at ca. 2.2 and 1.77 eV) has a lifetime of around 11.6 μs for MCH, 8.4 μs for PhMe, and 1.5 μs for 2MeTHF. By comparing with the photoluminescence decay, we assigned these species to be responsible for the delayed fluorescence (DF). As previously discussed by us,^12^ the DF component of **DMAC-TRZ** is a radiative decay from ^1^CT, mediated by the triplet excited states. Therefore, the *X* component PIA signal is related to an induced absorption transition from ^1^CT to upper singlet excited states (S_*n*_), and this transition in nsTA measurement decays at the same rate as that of the PL itself. As discussed above, the nature of the upper singlet excited states is identified by the unchanged energy transition at different solvent polarities. This confirms that the energy transitions observed in the PIA *X* channel induced absorption are ^1^CT_1_ → ^1^CT*_n_* and ^1^CT_1_ → ^1^CT_*m*_ transitions. In the *Y* channel, the peak of induced absorption presented a lifetime of around 0.14 ms in both MCH (−Δ*T*/*T* = 2.5 × 10^–4^) and PhMe (−Δ*T*/*T* = 0.5 × 10^–4^) solvents, which is assigned to be a transition from the QA conformer ^3^LE_1_ to upper triplet states (^3^LE_*n*_) based on the strong vibronic character of the transition. In 2MeTHF, this longer lifetime *Y* channel signal is significantly weaker (−Δ*T*/*T* = 0.1 × 10^–4^) because the ^3^CT state of QE becomes the lowest triplet excited state of the molecule, relaxed energetically below the ^3^LE state, which quenches the ^3^LE QA state.

In summary of this section, the proposed energy ordering of the excited states of **DMAC-TRZ** in MCH, PhMe, and 2MeTHF is shown in Figure 2. Clearly, by increasing the solvent polarity, a reordering of the excited states is observed, manifesting in a total change of the long lifetime signal and monotonic decrease of band intensity with increasing polarity observed in the short lifetime signals. In the higher polarity medium, the significant reduction of *f* decreases the photoinduced absorption signal intensity as well as relaxes the absolute energies of all CT states, so that the lowest energy ^3^CT triplet state becomes the lowest overall triplet excited state, quenching the population of the ^3^LE state. Consequently, the well-structured long-lifetime PIA band at 2.2 eV is observed only in MCH. Effectively, an alternative perspective on the mechanism is that, as the polarity increases, the lowest triplet excited state of the QA conformer, with its mixed LE/CT character, enhances the contribution of CT and relaxes to lower energies, driving the conformational change that impacts the populations of the different triplet states/conformers. This reordering of excited states then has a direct effect on the rISC rate; in MCH and PhMe, the rISC rates are 6 × 10^4^ and 27 × 10^4^ s^–1^, respectively, whereas in 2MeTHF, it rises considerably to 1.6 × 10^6^ s^–1^ (Table 1). The rISC rate values were estimated from the photoluminescence lifetimes, which was applied in a kinetic modeling according to Haase et al.^17^ Thus, we clearly observe that, as the energy ordering of the different triplet states changes, the rISC rate can increase by 2 orders of magnitude, achieving a very high rISC rate in 2MeTHF. As observed from the delayed fluorescence decays (Figure S4), in 2MeTHF, the PF/DF ratio becomes much smaller; i.e., DF starts to dominate much earlier, completely in line with the fastest *k*_rISC_. In 2MeTHF, we assume that ^1^CT relaxes below ^3^LE and ^3^CT is the lowest triplet state. Thus, as ^3^CT and ^3^LE are vibronically coupled, the subsequent ^3^LE crossing to ^1^CT is downhill in energy, resulting in an overall faster rISC rate. The increase in rISC rate when ^3^CT relaxes below ^3^LE is in good agreement with the theoretical predictions of Gibson and Penfold.^10^ We also note that ^3^CT is lower in energy, competing with increasing nonradiative decay via the energy gap law, quenching some of the population of the ^3^CT state, causing the DF decay rate to become the product of rISC and nonradiative decay rates.

To further analyze the broad negative PIA signal (−Δ*T*/*T*) in the *X* channel, we performed ultrafast transient absorption (fsTA) on the subpicosecond (top panel in Figure 4) as well as nanosecond time resolution (bottom panel in Figure 4). Figure 4a shows an instantaneous vibronic structured induced absorption of **DMAC-TRZ** in MCH, with maxima at ca. 1.7 and 1.9 eV and the onset of a further feature at 1.5 eV. While the two peaks at 1.7 and 1.9 eV have similar decay lifetimes, the 1.5 eV TA feature has a different kinetic (Figure S12). This combined with the appearance of an isosbestic point at 1.6 eV suggests that two different species are present with overlapping induced absorption. As mentioned before, the QA and QE conformer of **DMAC-TRZ** can be seen in nonpolar solvents. The QE conformer has a long fluorescence lifetime, and so, we attribute the vibronic absorption band with maxima of 1.7 and 1.9 eV to this conformer. While the very short lifetime QA conformer contribution gives rise to the short-lived photoinduced absorption feature at 1.5 eV, which decays within 20 ps, this rapid decay of the QA conformer is likely due to a singlet excited state interconversion between the QA to the QE conformer. Despite a very small QA population observed in nonpolar solvent,^26^ as seen in Figure S5, strong absorption transitions are expected for this conformer due to its stronger *f* when compared to the QE conformer. Rapid decay at 1.5 eV of the induced absorption and the isosbestic point at 1.6 eV indicate interconversion between these two stable conformers. However, no evident growth of the intensity of the photoinduced absorption feature at 1.7 and 1.9 eV coming from a ^1^CT transition of the QE conformer was observed. This corroborates no significant increase in the QE population from the QA interconversion, as its relative population is known to be very small. Interestingly, the singlet excited state interconversion of QA to QE conformer is highly dependent on the excitation wavelength. As shown in Figure S5b, the excess energy provided by the higher energy excitation accelerates the interconversion between the two conformers, leading to no detected emission from the QA conformer by exciting at 300 and 280 nm. Even though we observed interconversion of the singlet excited state from the QA conformer, in the triplet excited state, the picture is different. The triplet excited state of the QA conformer is the lowest triplet excited state in MCH and is mainly populated via Dexter energy transfer from the QE conformer. Its longer lifetimes (around a hundred microseconds) suggest that the triplet excited state of the QA conformer does not interconvert to the QE conformer. We point out that these solutions have a relatively high concentration of 0.8 mM, which leads to a decrease in the distance between both conformers and an increased probability of collisional Dexter transfer. Furthermore, as discussed above, the broad induced negative absorption (−Δ*T*/*T*) measured in the quasi-CW photoinduced absorption comes from a ^1^CT transition, which based on the transient absorption has strong mixed LE/CT character. Consequently, in a nonpolar solvent MCH, it has a higher contribution of LE character, consistent with the strong vibronic structure observed for this transition.

**Figure 4:**
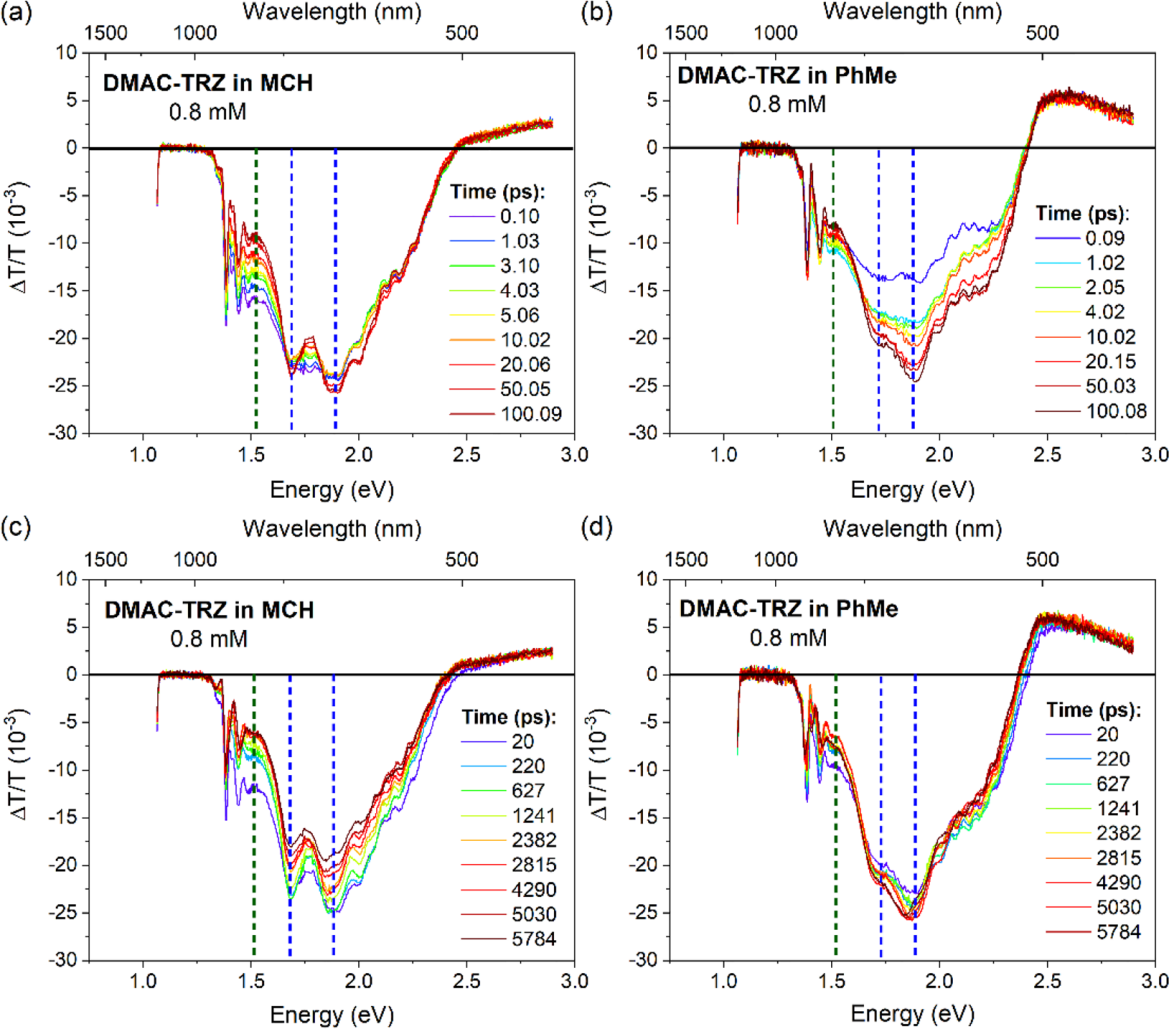
Picosecond transient absorption spectra (top panel) and nanosecond transient absorption spectra (bottom panel) of **DMAC-TRZ** in (a) MCH and (b) PhMe solutions at 0.8 mM. λ_exc_ (pump) = 343 nm (which direct excites QA and QE conformers).

While in MCH little reorganization of the solvent dipoles around the excited **DMAC-TRZ** is expected, in a polar solvent, such as PhMe, this effect is much larger. Figure 4b (and kinetic traces, Figure S12) shows that the vibronic structured band at 1.7 and 1.9 eV displays an apparent intensity build-in time, ca. 17 ps. This could be due to solvent reorganization upon excitation; an initial population of ^1^CT is generated, which induces reorganization of the solvent dipole moment of the surrounding solvent shell. In this case, solvent reorganization stimulates a change in the character of these excited states, which consequently increases *f* of the ^1^CT_1_ → ^1^CT_*n*_ transition. We detect the change in *f* as the build-in within 17 ps in the transient absorption signal, although this seems rather slow for such a process. It is expected that increasing solvent polarity enhances the CT character of the excited state while reducing *f*. This is because the excited state character becomes more distinct than the ground state reducing the electronic coupling between the two. In the context of the transient absorption, the lowest excited state is not a ground state but ^1^CT. Therefore, as solvent polarity increases, both excited states become more similar in nature, consequently resulting in an increased *f* for this upper state transition (^1^CT_1_ → ^1^CT_*n*_). Noteworthy, there is no increase observed in the PL/SE intensity, suggesting that the population of the CT_1_ excited state remains the same, indicating that the increase in intensity of the PIA is not caused by an increase in the excited state population. Further, the QA contribution (induced absorption at 1.5 eV) becomes less pronounced in higher solvent polarity, as already reported by us.^26^

In the longer time scale fsTA (bottom panel in Figure 4c), we simply observed a decay in the transient absorption band of **DMAC-TRZ** in MCH. This is a result of a competition between the induced absorption of the ^1^CT to ^1^CT_*n*_/^1^CT_*m*_ and the radiative decay of the ^1^CT state. By fitting the decay curve (Figure S12), we estimated the lifetime around 18 ns, which is in good agreement with the 13.6 ns lifetime of the prompt component in the PL decay curve (Table 1). However, we observed no decay in the transient absorption within the 6 ns time window of our PIA measurement in PhMe (Figure 4d). This indicates that the radiative decay of this state has a much longer lifetime, which is expected for **DMAC-TRZ** from emission measurements to be around 21 ns in PhMe. Thus, the ultrafast transient absorption not only confirms the excited states responsible for photoinduced absorption signal in the *X* channel from quasi-CW photoinduced absorption but also verifies the contributions of two stable conformers. With the ultrafast transient absorption, we also gained insights into the dynamics of the solvent and/or molecular reorganization induced by the polarity and how it can strongly affect the *f* on the singlet upper excited state transitions.

To conclude, we used photoinduced absorption techniques to probe the excited states of the TADF molecule **DMAC-TRZ** in solution. By performing quasi-CW photoinduced absorption and transient absorption, we identified both the transitions and the nature of the electronic excited states involved. The photoinduced absorption signals are highly dependent on the solvent polarity as expected, which have stronger oscillator strength in MCH. Surprisingly, the different solvent polarity does not change the peak positions, implying that the transitions are between upper and lower excited states of the same orbital character, i.e., CT to CT in this case. In the Y channel of quasi-CW photoinduced absorption in MCH, we observed a strong and vibronic structured absorption band from the ^3^LE to upper triplet states (^3^LE_*n*_). This transition ^3^LE_1_ → ^3^LE_*n*_ is quenched by increasing solvent polarity, clearly indicating that ^3^CT becomes the lowest triplet state in PhMe and 2MeTHF.

Using ultrafast transient absorption measurements, we uncovered the nature of the broad photoinduced band observed in the *X* channel. Isosbestic points are observed in MCH solution, a signature of the presence of two species with overlapping induced absorption, originating from the two stable conformers **DMAC-TRZ**, axial and equatorial conformers previously reported. The QA conformer rapidly decays within 20 ps, indicating a singlet excited state interconversion from the QA to the QE conformer. By increasing the polarity of the solvent, a smaller contribution of the QA conformer is expected; thus, less pronounced effect of the singlet excited state interconversion between these two stable conformers was observed.

Moreover, the higher polarity of PhMe induces solvent reorganization, as evidenced by a significant build-in of the intensity of the PIA band within 17 ps but no change in the intensity of the PL/SE band. This indicates that the excited state population does not increase, so the increase in induced absorption intensity must be due to a change in the oscillator strength of the transition giving rise to the induced absorption band.

## Supporting Information Available

General experimental procedures, molecular structure of **DMAC-TRZ**, additional experimental measurements, and calculated and proposed energy diagrams (PDF)
