# Supplementary material for: Exploring the Early Time Behavior of the Excited States of an Archetype Thermally Activated Delayed Fluorescence Molecule
Source: J Phys Chem Lett. 2024 Feb 7;15(6):1734–40. doi: 10.1021/acs.jpclett.4c00030 (PMC10875657; doi:10.1021/acs.jpclett.4c00030)
Supplement: Supplementary file 1 — jz4c00030_si_001.pdf [file jz4c00030_si_001.pdf]

Supporting information for:

Exploring the early time behaviour of the excited states of an archetype thermally activated delayed fluorescence molecule

*Larissa G. Franca,[a] Andrew Danos,[a] Rishabh Saxena,[b] Suman Kuila,[a] Kleitos Stavrou,[a] Chunyong Li,[a] Stefan Wedler,[b] Anna Köhler,[b] Andrew P. Monkman [a,]**

[a] Department of Physics, Durham University, South Road, Durham, DH13LE, United Kingdom

[b] Soft Matter Optoelectronics and Bavarian Polymer Institute (BPS), Universitätsstrasse 30, University of Bayreuth, Bayreuth, 95440, Germany

Experimental section:

a. Sample preparation

Optical characterization of **DMAC-TRZ** was performed in three different solvents: methylcyclohexane (MCH), toluene (PhMe) and 2-methyltetrahydrofuran (2MeTHF). Solutions with 20 μM concentration were prepared for absorption spectra. While for the photoluminescence spectra, quasi-CW photoinduced absorption and transient absorption, the solutions were prepared at a concentration of 0.8 mM and degassed by five freeze−pump−thaw cycles.

b. Photoluminescence measurements

Absorption spectra were recorded with UV-3600 double beam spectrophotometer (Shimadzu). Photoluminescence (PL) spectra were recorded using a QePro compact spectrometer (Ocean Optics). Time-resolved photoluminescence spectra were recorded using an ultra-fast 4 PICOS iCCD camera (Stanford Computer Optics) with a pulsed (10 Hz) Nd:YAG laser (EKSPLA-SL312) excitation source at 355 nm. Lifetimes were obtained by fitting with monoexponential functions using Magicplot software.

c. Quasi CW photoinduced absorption

The quasi-CW PIA measurements were performed using a 375 nm pump beam (Vortran Stradus 375-60) modulated at 173 Hz, with a continuous laser driven white light

source (Energetiq EQ-99X) as the probe. The probe beam was then passed through a Bentham TM300 monochromator and incident on a Si and Ge detector connected to the Signal Recovery dual channel 7225 digital lock-in amplifier that also provides the reference frequency modulation for the pump laser.

d. Transient photoinduced absorption

Guided by the PIA spectra, nanosecond kinetics and the absolute signs of the induced absorption signals were determined at wavelengths of interest. The broadband output of a laser driven light source (Energetiq EQ-99) was focused with aluminium parabolic mirrors (Thorlabs) into a spot of ~ 4 mm diameter on the 1 cm sample cuvette, acting as a probe beam. The output of a Nd:YAG laser (EKSPLA, ~ 7 ns pulse width, 355 nm, 500 Hz repetition rate) was aligned with this spot in a quasi-coaxial beampath, passing directly from the sample into a beamdump. The transmitted probe beam was refocussed into the slits of a Bentham TMc300 monochromator, and received by either a balanced Si (Femto HBPR-200M-30K-Si) or single-element InGaAs (Femto HCA-S-200M-IN) photodiode. The diode output was optionally amplified (Femto DHPVA-201) and read using an oscilloscope (Agilent Infiniium 1 GHz) triggered by the pump laser. In this way changes in diode output following the pump are directly proportional to ΔT at the set wavelength, and have the same decay kinetics.

For measurements at visible wavelengths, significant oscillations of the probe beam (~10% of total intensity, oscillating at 42 kHz and arising from pulsed laser driving of the output plasma) were addressed in hardware rather than through averaging. Additionally, contributions from visible pump-induced emission were also collected at these wavelengths. Using the balanced Si diode, one arm was coupled to the monochromated probe beam emerging from the sample. Using an optical fibre and variable filter wheel, the other arm was coupled to a portion of the pre-sample probe beam (also coherently containing the intensity variations, but none of the sample response), using reflection from a quartz plate. With the pump beam blocked the filter wheel was used to balance the intensities of the probe beam in the two signal channels, leading to a balanced output with the probe beam signal and oscillations cancelled. With the pump beam then unblocked, the transient absorption signal could be collected. This approach means however that absolute measurements of T (the denominator in $\Delta T/T$) become inaccessible, although with greatly improved ability to measure the kinetics of ΔT. Emission from the sample was then also collected using the same oscilloscope settings by simply blocking the probe

beam. In some cases, the vertical scale of the oscilloscope was set deliberately to capture the delayed response with high fidelity, leading to apparent saturation of the prompt signal (e.g. **Figure 3c**).

e. Ultrafast photoinduced absorption

The laser is PHAROS from Light Conversion (wavelength: 1030 nm, pulse duration: 180 fs). Part of the output is used to do third harmonic generation (THG), which produces 343 nm output. The 343 nm output is used to pump our samples. Another part of the original laser output pumps a 2 mm sapphire plate to generate a white light continuum (WLC). This WLC is used to probe the dynamics of the excited states.

Chemical structure:

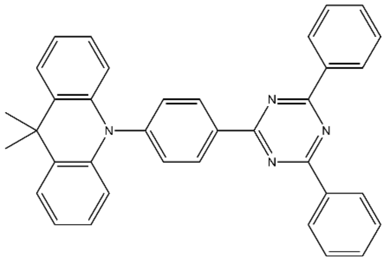

Figure S1: Molecular structure of the emitter molecule (**DMAC-TRZ**).

Photophysical properties:

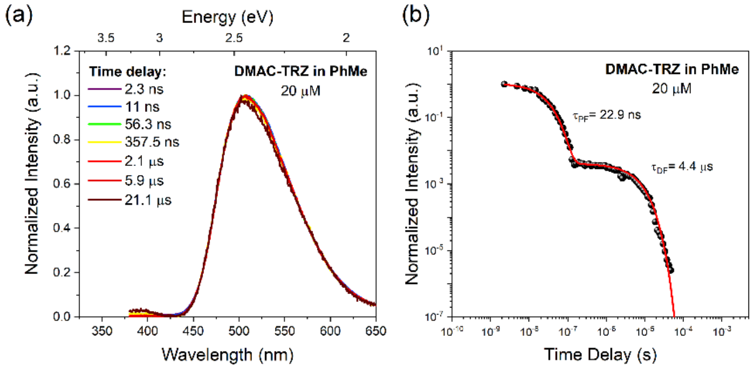

Figure S2: Time-resolved emission spectra and kinetic decays of **DMAC-TRZ** in PhMe solutions at 20 µM concentration. All measurements were performed in degassed solutions at room temperature, using a 355 nm excitation.

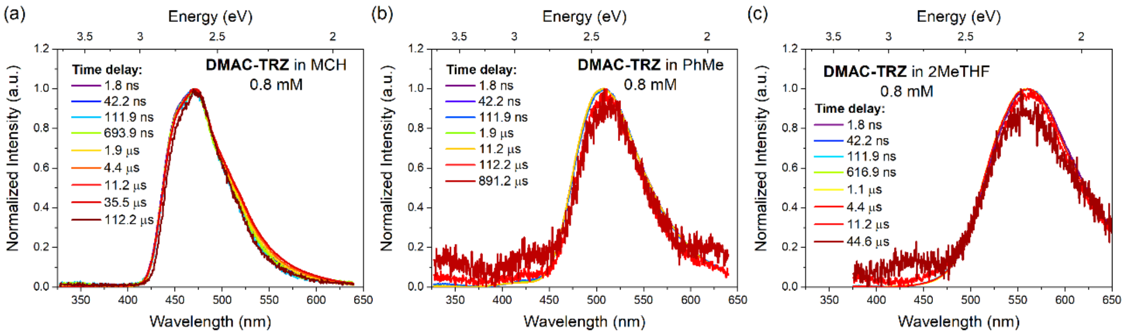

Figure S3: Time-resolved emission spectra of **DMAC-TRZ** in (a) MCH, (b) PhMe and (c) 2MeTHF solutions, at 0.8 mM concentration. All measurements were performed in degassed solutions at room temperature, using a 355 nm excitation source.

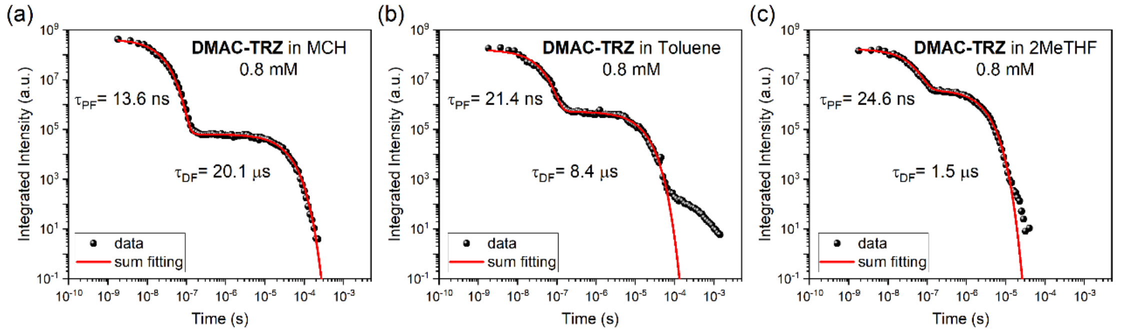

Figure S4: Kinetic decays of **DMAC-TRZ** in (a) MCH, (b) PhMe and (c) 2MeTHF solutions, at 0.8 mM concentration. All measurements were performed in degassed solutions at room temperature, using a 355 nm excitation source. The data are fitted using a biexponential function.

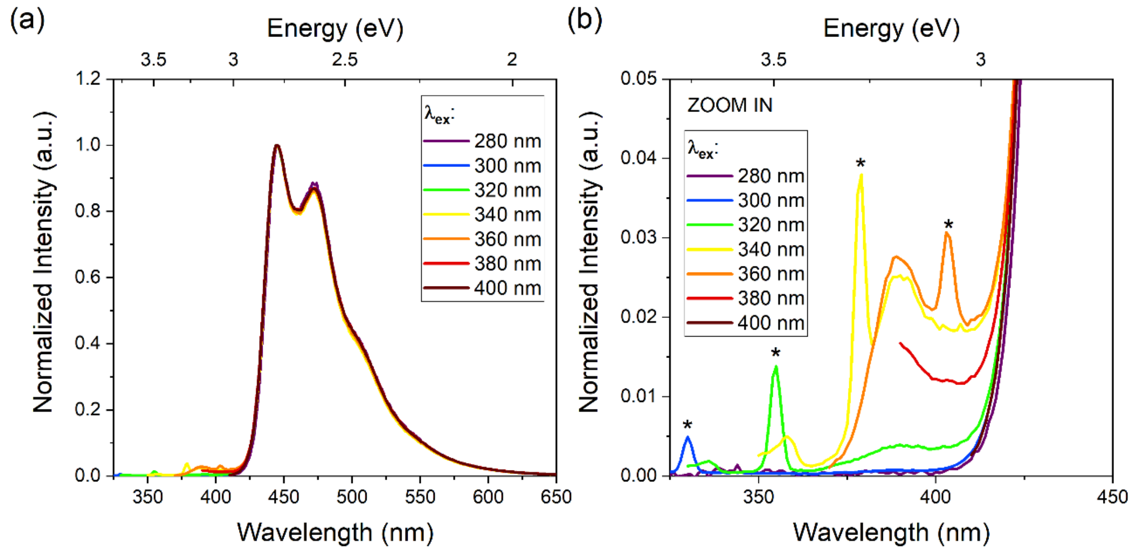

Figure S5: (a) Photoluminescence spectra of **DMAC-TRZ** in MCH solution excited at different wavelengths, at 20 µM concentration. (b) Zoom in on the photoluminescence spectra to highlight the QA conformer dependence on the excitation wavelength. *Solvent Raman peaks.

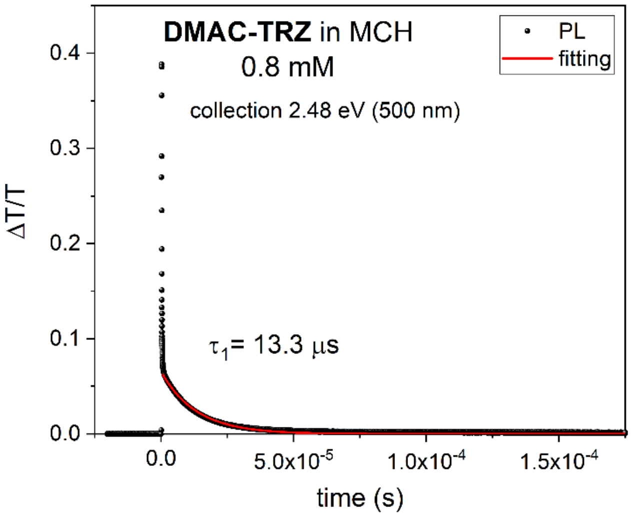

Figure S6: Transient photoinduced absorption of **DMAC-TRZ** in MCH at 0.8 mM concentration. The measurements were collected at 2.48 eV (500 nm), with the pump only (PL). The data are fitted using a monoexponential function.

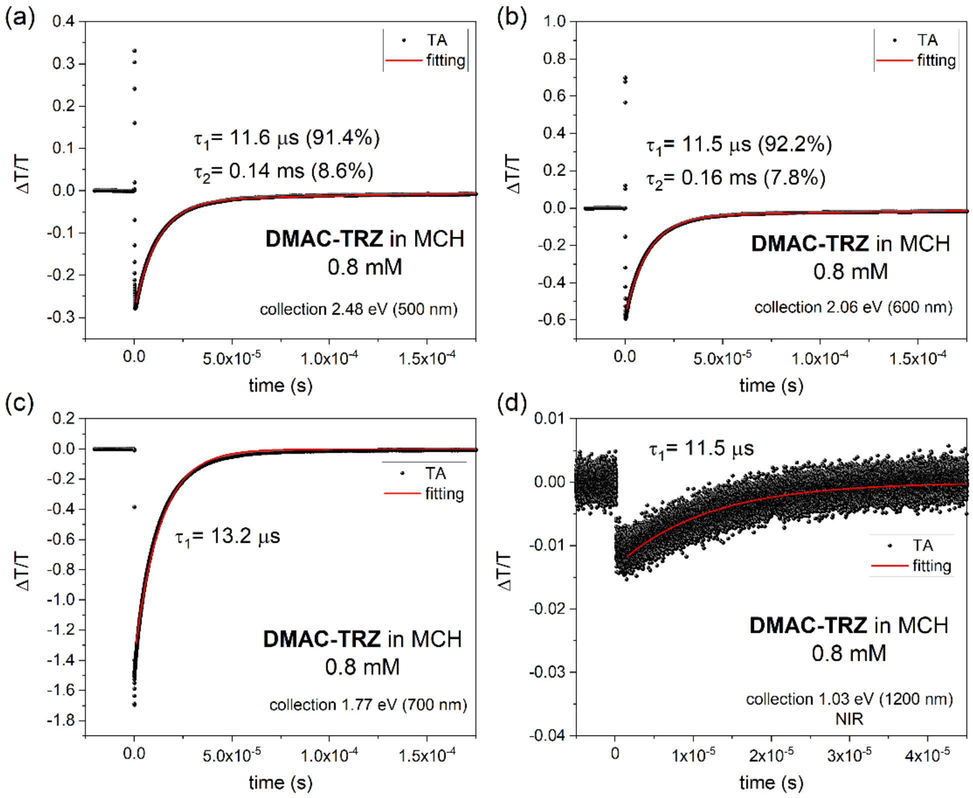

Figure S7: Transient photoinduced absorption of **DMAC-TRZ** in MCH at 0.8 mM concentration. The measurements were collected at: (a) 2.48 eV (500 nm), (b) 2.06 eV (600 nm), (c) 1.77 eV (700 nm) and (d) 1.03 eV (1200 nm), with the pump and probe (TA). The data are fitted using monoexponential and biexponential functions.

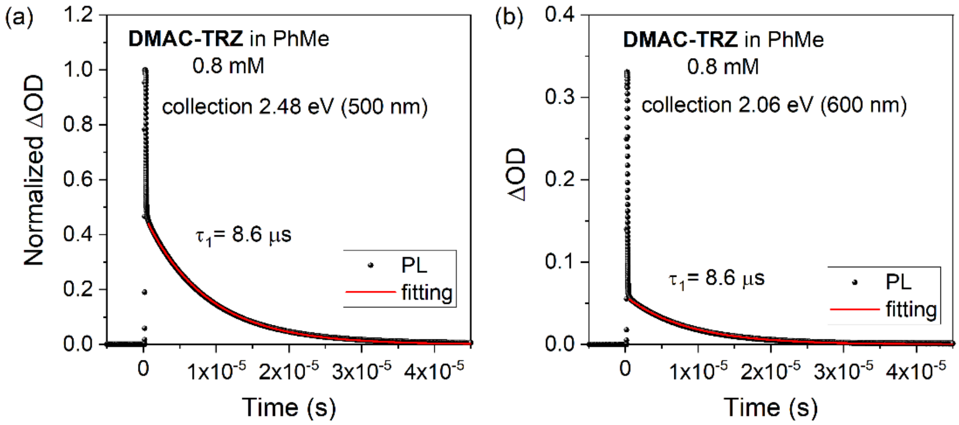

Figure S8: Transient photoinduced absorption of **DMAC-TRZ** in PhMe at 0.8 mM concentration. The measurements were collected at: (a) 2.48 eV (500 nm) and (b) 2.06 eV (600 nm), with the pump only (PL). The data are fitted using a monoexponential function.

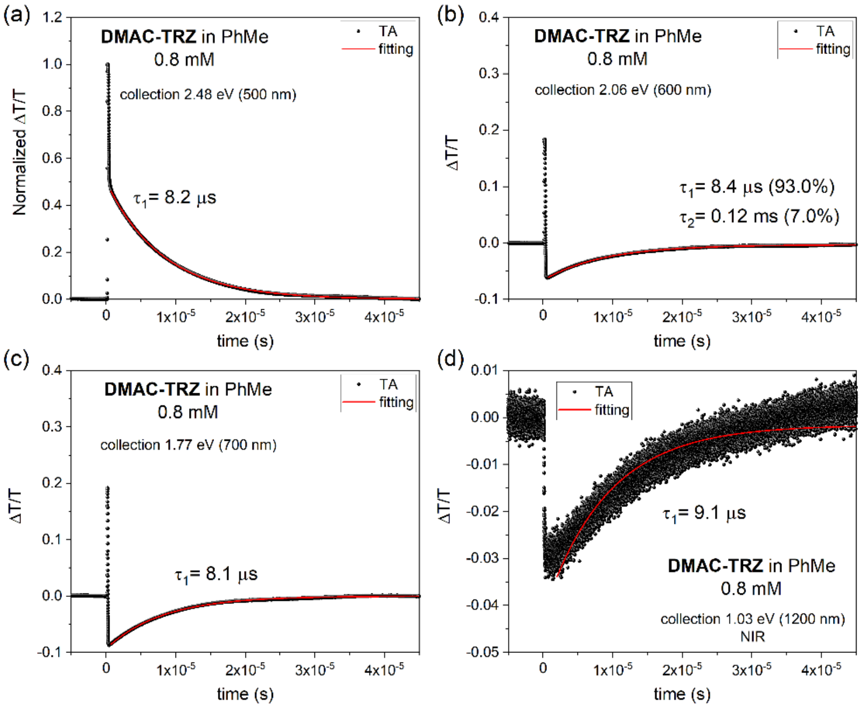

Figure S9: Transient photoinduced absorption of **DMAC-TRZ** in PhMe at 0.8 mM concentration. The measurements were collected at: (a) 2.48 eV (500 nm), (b) 2.06 eV (600 nm), (c) 1.77 eV (700 nm) and (d) 1.03 eV (1200 nm), with the pump and probe (TA). The data are fitted using monoexponential and biexponential functions.

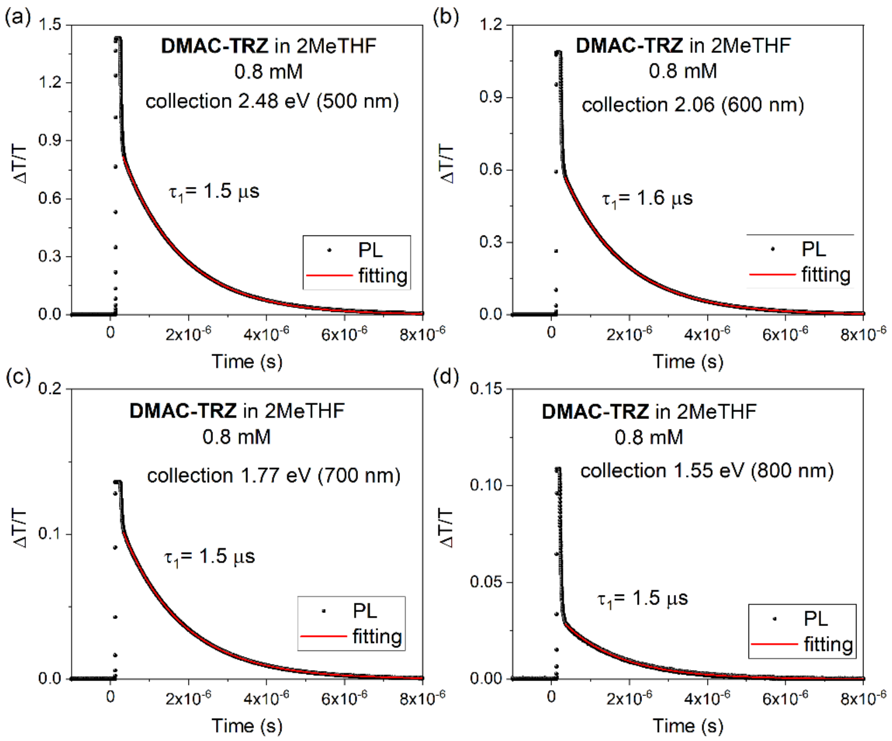

Figure S10: Transient photoinduced absorption of **DMAC-TRZ** in 2MeTHF at 0.8 mM concentration. The measurements were collected at: (a) 2.48 eV (500 nm), (b) 2.06 eV (600 nm), (c) 1.77 eV (700 nm) and (d) 1.55 eV (800 nm), with the pump only (PL). The data are fitted using a monoexponential function.

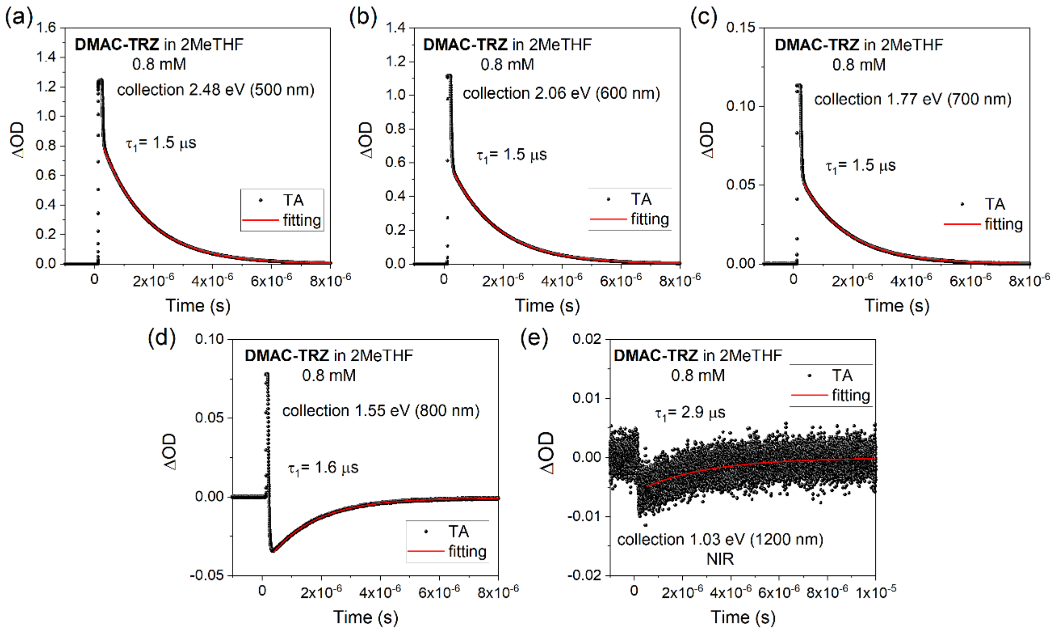

Figure S11: Transient photoinduced absorption of **DMAC-TRZ** in 2MeTHF at 0.8 mM concentration. The measurements were collected at: (a) 2.48 eV (500 nm), (b) 2.06 eV (600 nm), (c) 1.77 eV (700 nm), (d) 1.55 eV (800 nm) and (e) 1.03 eV (1200 nm), with the pump and probe (TA). The data are fitted using monoexponential function.

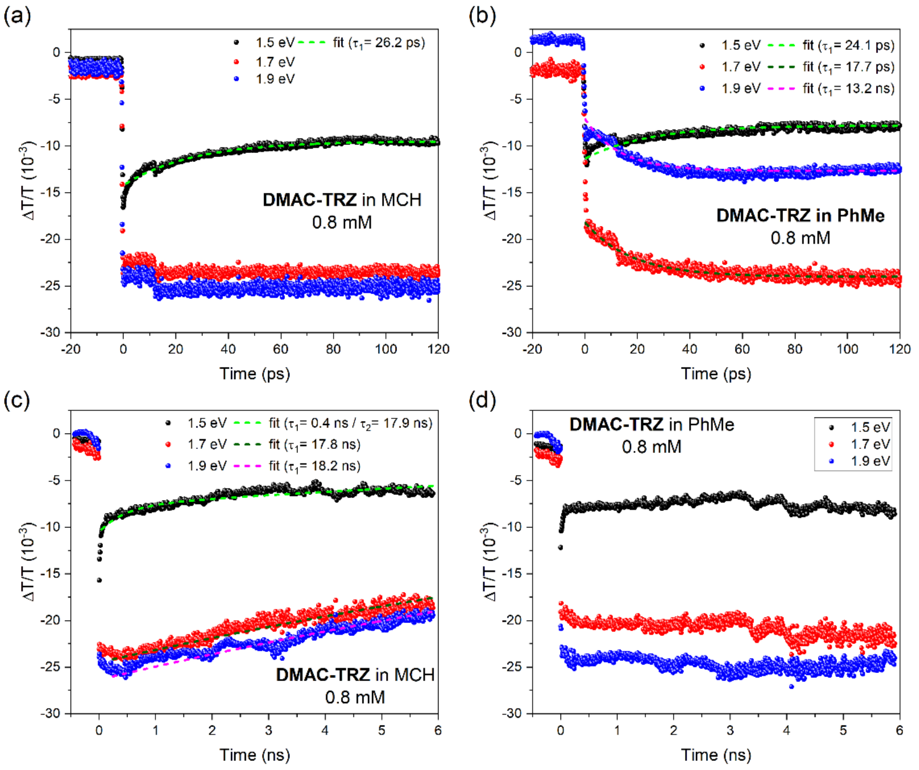

Figure S12: Picosecond transient absorption decay curves (top panel) and nanosecond transient absorption decay curves (bottom panel) of **DMAC-TRZ** in MCH (a and c) and PhMe (b and d) solutions at 0.8 mM., collected at 1.5, 1.7 and 1.9 eV. The data are fitted using monoexponential and biexponential functions.

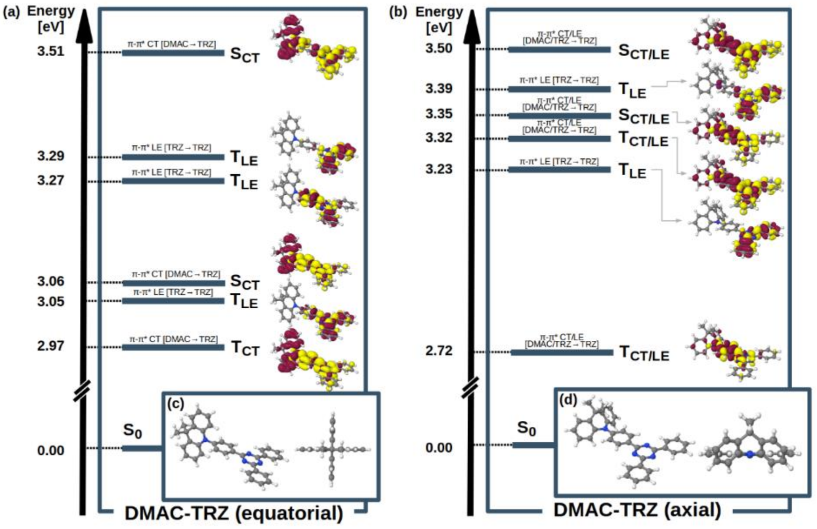

Figure S13: Calculated vertical DFT/MRCI excitation energies and state characteristics of the a) QE and b) QA conformers of **DMAC-TRZ** at the respective ground-state geometry in methylcyclohexane solution. The QE conformer is favored over the QA conformer by 0.19 eV including ZPVE corrections. A loss of electron density w.r.t. the electronic ground state is indicated in red, a gain in yellow. Geometric structures of the c) quasi-equatorial and d) quasi-axial conformers of **DMAC-TRZ** in the electronic ground state. Data reported by *Monkman et al.*[1]

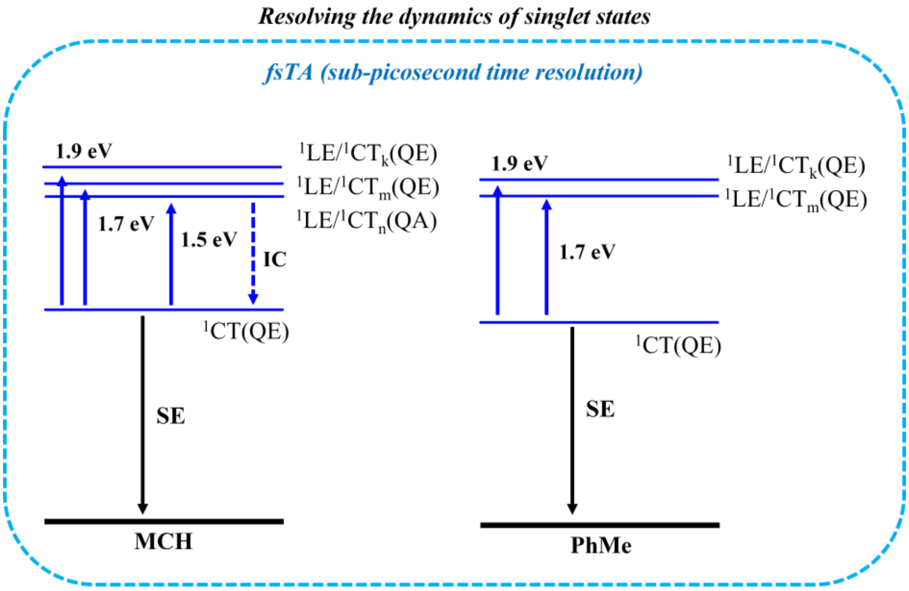

Figure S134: Proposed excited state energy diagram, representing the spectra obtained by the ultrafast transient absorption of **DMAC-TRZ** in MCH and PhMe.

(1) Stavrou, K.; Franca, L. G.; Böhmer, T.; Duben, L. M.; Marian, C. M.; Monkman, A. P. Unexpected Quasi-Axial Conformer in Thermally Activated Delayed Fluorescence DMAC-TRZ, Pushing Green OLEDs to Blue. *Adv. Funct. Mater.* **2023**, *33* (25), 2300910.
